# The audience effect in adolescence depends on who's looking over your shoulder

**DOI:** 10.1016/j.adolescence.2015.05.003

**Published:** 2015-08

**Authors:** Laura K. Wolf, Narges Bazargani, Emma J. Kilford, Iroise Dumontheil, Sarah-Jayne Blakemore

**Affiliations:** a UCL Institute of Cognitive Neuroscience, 17 Queen Square, London WC1N 3AR, UK; b UCL Department of Neuroscience, Physiology and Pharmacology, Gower St, London WC1E 6BT, UK; c Department of Psychological Sciences, Birkbeck, University of London, Malet Street, London WC1E 7HX, UK

**Keywords:** Adolescence, Peer influence, Audience effect, Reasoning, Development

## Abstract

Adolescents have been shown to be particularly sensitive to peer influence. However, the data supporting these findings have been mostly limited to the impact of peers on risk-taking behaviours. Here, we investigated the influence of peers on performance of a high-level cognitive task (relational reasoning) during adolescence. We further assessed whether this effect on performance was dependent on the identity of the audience, either a friend (peer) or the experimenter (non-peer). We tested 24 younger adolescent (10.6–14.2 years), 20 older adolescent (14.9–17.8 years) and 20 adult (21.8–34.9 years) female participants. The presence of an audience affected adolescent, but not adult, relational reasoning performance. This audience effect on adolescent performance was influenced by the participants' age, task difficulty and the identity of the audience. These findings may have implications for education, where adolescents often do classwork or homework in the presence of others.

## Introduction

There is a long history of social psychology studies on the effects of the presence of another person on performance – predominantly in adults (Aiello & Douthitt, 2001; Zajonc, 1965). These effects, known as social facilitation, or more specifically, audience effects, describe the influence of an audience on performance measures, such as accuracy and response time (RT). However, few developmental studies have investigated the audience effect (Meddock, Parsons, & Hill, 1971; Newman, Dickstein, & Gargan, 1978; Quarter & Marcus, 1971). Social information is thought to have particularly high salience during adolescence, in particular in the context of relationships with peers (Blakemore & Mills, 2014). The aim of the current study was to investigate the development of the audience effect between adolescence and adulthood, and to examine to what extent the audience effect was influenced by the identity of the observer (peer versus non-peer), and the difficulty of the task.

### Peer influence during adolescence

During the transition from childhood to adolescence, relationships with peers become increasingly elaborate, more personal and emotional (Brown, 2004) and interactions with peers dominate adolescents' social environment, with American adolescents spending more than half of their awake-time with peers (Csikszentmihalyi & Larson, 1984). Adolescent decision-making is also particularly influenced by their peers (Brechwald & Prinstein, 2011; Brown, 2004). Evidence of increased sensitivity to peer influence during adolescence comes from both experimental and questionnaire data. Experimental studies have demonstrated that adolescents are particularly sensitive to the presence of peers when making risky and reward-related choices (Chein, Albert, O'Brien, Uckert, & Steinberg, 2011; O’Brien, Albert, Chein, & Steinberg, 2011; Reynolds, MacPherson, Schwartz, Fox, & Lejuez, 2013; Smith, Steinberg, Strang, & Chein, 2014). For example, when performing a driving video game, adolescents (13–16 years) took more risks when being observed by peers relative to when alone, while adults' risk-taking was not affected by the presence of peers (Gardner & Steinberg, 2005). If the increased sensitivity to the presence of peers found in risky and reward-related decision-making extends to other domains, adolescents might also display greater sensitivity to audience effects than adults in cognitive task performance.

Peer influence may change within the period of adolescence: in an experimental study adolescents (aged 11.9–15.8), and in particular younger adolescents (aged 11.9–13.9), were shown to be hypersensitive to social exclusion (Sebastian, Viding, Williams, & Blakemore, 2010), suggesting a greater sensitivity of younger adolescents to the social context. Research using the resistance to peer influence (RPI) questionnaire has demonstrated that resistance to peer influence is greater in adults than in younger adolescents, with the most pronounced increase occurring between 14 and 18 years (Steinberg & Monahan, 2007). Consistent with the experimental data described above, this suggests younger adolescents might be more influenced by the presence of a peer. However, results from another questionnaire-based study demonstrated that 15–18 year-olds reported increased levels of fear of social evaluation relative to 12–14 year-olds and 8–11 year-olds (Westenberg, Drewes, Goedhart, Siebelink, & Treffers, 2004), suggesting older adolescents might be more concerned about being evaluated by their peers. The present study included participants aged 10–17 years, enabling us to investigate potential developmental differences within adolescence, although, based on mixed evidence from previous research; it was unclear whether younger or older adolescents would show greater audience effects. As participants with lower resistance to peer influence may be more sensitive to the presence of a peer audience, we also investigated whether greater audience effects were associated with lower self-reported resistance to peer influence.

### Adolescent sensitivity to a peer audience

In a recent neuroimaging study, participants aged 8–22 years were told they would sometimes be watched by a peer via a camera while lying in the scanner (Somerville et al., 2013). When adolescents thought they were being observed by a peer, they showed higher autonomic arousal as measured by skin conductance, relative to both children and adults. Self-reported embarrassment and activation in the medial prefrontal cortex - a key region of the social brain (Frith & Frith, 2007) – were also elevated in adolescence relative to late childhood (Somerville et al., 2013). In accordance with findings from peer influence studies, this suggests that adolescents may be particular sensitive to being observed by a peer audience.

In an electroencephalography study, Kim, Iwaki, Uno, and Fujita (2005) investigated whether the presence of a friend influenced performance and error-related negativity (ERN; a negative deflection occurring shortly after an error has been committed) in a go/no-go task in 7–11 year-olds. There was no effect on behaviour, however participants showed increased ERN-amplitudes in the presence of a friend, relative to being alone, indicating that 7–11 year-olds are already sensitive to the presence of peers when performing a cognitively demanding task. There is little experimental research on how peer influence affects cognitive performance, particularly during adolescence. In a sample of 9–14 year-olds with behavioural problems, performance in a relational reasoning task decreased in the presence of a classmate (Bevington & Wishart, 1999). However, in this sample it is difficult to disentangle the effects of the presence of the peer on performance from the effects of disruptive behaviour. Previous studies have not investigated whether typically developing adolescents show increased sensitivity to the presence of a peer audience during cognitive task performance.

### Influence of the identity of the observer

Previous peer influence studies have compared peer observation with no observation (Gardner & Steinberg, 2005; O'Brien et al., 2011), making it impossible to attribute effects to the specific presence of a peer rather than to general effects of the presence of another person. The present study manipulated audience across three levels: peer audience (the participant's friend), non-peer audience (the experimenter) and no audience. By comparing peer versus non-peer observation conditions, we were attempting to control for any general (e.g. distracting) effects of having someone present while performing a task. With this design we tested the hypothesis that adolescents would be particularly sensitive to being observed by a peer relative to being observed by a non-peer. In addition, friendship quality between the participant and their friend may differ between age groups and thus we collected self-reported measures of friendship to control for potential developmental differences.

### Choice of paradigm and influence of task difficulty

To investigate developmental differences in the audience effect, i.e. the effect of an observer on cognitive performance rather than decision making, we used a relational reasoning paradigm, which involves evaluating and integrating the relationships between multiple mental representations (Krawczyk, 2012). Examples of such relationships are analogies (Holyoak, 2012), in which a new piece of information is understood by comparing it to existing knowledge, which facilitates problem solving in novel situations and knowledge transfer across different contexts (Krawczyk, 2012). Relational reasoning has been found to be associated with mathematics performance, reading and academic knowledge (Ferrer, O'Hare, & Bunge, 2009; Träff, 2013).

The difficulty of relational reasoning problems can be quantified by the number of relations that need to be considered to solve them (Raven, 1941). To solve a 1-relational problem, variation along one dimension must be considered, while solving 2-relational (and higher) problems requires integrating two or more dimensions of variation. While children under 5 years can solve 0- and 1-relational problems, they fail to solve 2-relational (or higher) problems (Halford, 1984). Relational reasoning continues to improve in late childhood and throughout adolescence (Crone et al., 2009; Dumontheil, 2014; Dumontheil, Houlton, Christoff, & Blakemore, 2010; Rosso, Young, Femia, & Yurgelun-Todd, 2004; Wendelken, O'Hare, Whitaker, Ferrer, & Bunge, 2011). As a secondary aim of this study, we investigated developmental differences in relational reasoning abilities, predicting improved performance with age in higher order relational trials.

Studies of the audience effect in adults have found that performance usually improves when participants are observed in simple and well-learned tasks, and worsens in complex or learning tasks (Bond & Titus, 1983). However, there does not seem to be a general agreement on what classifies a task or task-level as ‘simple’ or ‘complex’, particularly as a wide variety of tasks have been used to study social facilitation (Bond & Titus, 1983; Guerin, 1986). In their meta-analysis, Bond and Titus (1983) classified tasks according to their label in the original papers, rather than using a systematic rule to classify tasks as simple or complex across studies. We included two task-levels to assess the potential specificity of audience effects to different levels of task difficulty. However, as relational reasoning is usually considered a high-level, complex task, even at relatively low levels, we were agnostic as to whether we would find differential audience effects for the two difficulty levels.

## Methods

### Participants

Pairs of volunteers were recruited for this study and randomly assigned at the beginning of the study (using a coin-flip) to either the role of the *participant*, who would perform the task, or the *observer*, who would watch the *participant* in one session and subsequently evaluate the *participant's* performance. The term ‘volunteers’ refers to both the *participants* and the *observers*.

Forty-four pairs of adolescent and 20 pairs of adult friends took part. Pairs of friends (rather than unknown peers) were invited as it was hypothesised that adolescents might care more about what their friends think about them when performing a high-level cognitive task than what an unknown peer may think. This has not been systematically investigated in experimental peer influence studies; however there is evidence that adolescent smoking behaviour is influenced more by best friends than by social crowds (Urberg, 1992). Grouping of the adolescent *participants* was performed by a median split, resulting in a group of 24 younger adolescents (aged 10.6–14.2) and 20 older adolescents (aged 14.9–17.8) (median age of all adolescent *participants* = 14.2 years, see Table 1 for ages). Volunteers were recruited from the Greater London area. Adolescents were recruited from local schools and sports clubs and adults were recruited from local universities and the Science Museum. Most adolescents attended academically selective secondary schools and the majority of adults were university graduates. Volunteers were paid £8/hour for their time. Procedures were approved by the local Research Ethics Committee and all volunteers (or their parents/guardians) gave informed consent.

Due to sex differences in brain maturation (Herting, Maxwell, Irvine, & Nagel, 2012; Raznahan et al., 2011) only female volunteers were recruited to maximise sample-homogeneity. Additionally, in the experimenter-present condition, the observer (LKW) was also female, thus ensuring the sex of the observer was matched across the two observation conditions. Two adult *participants* were excluded from the analysis (one performed below chance and one verbalised her strategies during the task); thus the analysis included 24 younger adolescents, 20 older adolescents and 18 adults. Verbal IQ of *participants* was assessed with the vocabulary subtest of the Wechsler Abbreviated Scale of Intelligence (WASI, Wechsler, 1999), to check groups were matched in terms of cognitive ability.

### Questionnaire measures

*Participants* completed the resistance to peer influence questionnaire (RPI, Steinberg & Monahan, 2007), consisting of ten statement-pairs pertaining to peer influence. *Participants* chose which statement described them best and rated it as ‘really true’ or ‘sort of true’. Responses were coded on a four-point scale - high scores indicating greater resistance (Cronbach's alpha reliability-coefficient for the sample: α = 0.65). Volunteers completed the McGill Friendship Questionnaire–Friend's Function (MFQ-FF, Mendelson & Aboud, 1999): 30 questions assessed how much the friend fulfils friendship functions and were rated on a nine-point scale (from 0 to 8) – with high scores indicating greater friendship quality. For each volunteer-pair a combined score of *participant* and *observer* reported friendship quality was generated (α = 0.95).

### Experimental design

#### Relational reasoning task

Stimuli were presented with Cogent 2000 (www.vislab.ucl.ac.uk/Cogent/index.html) implemented in Matlab R2010b (Mathworks Inc., Sherborn, MA).

**Fig. 1:**
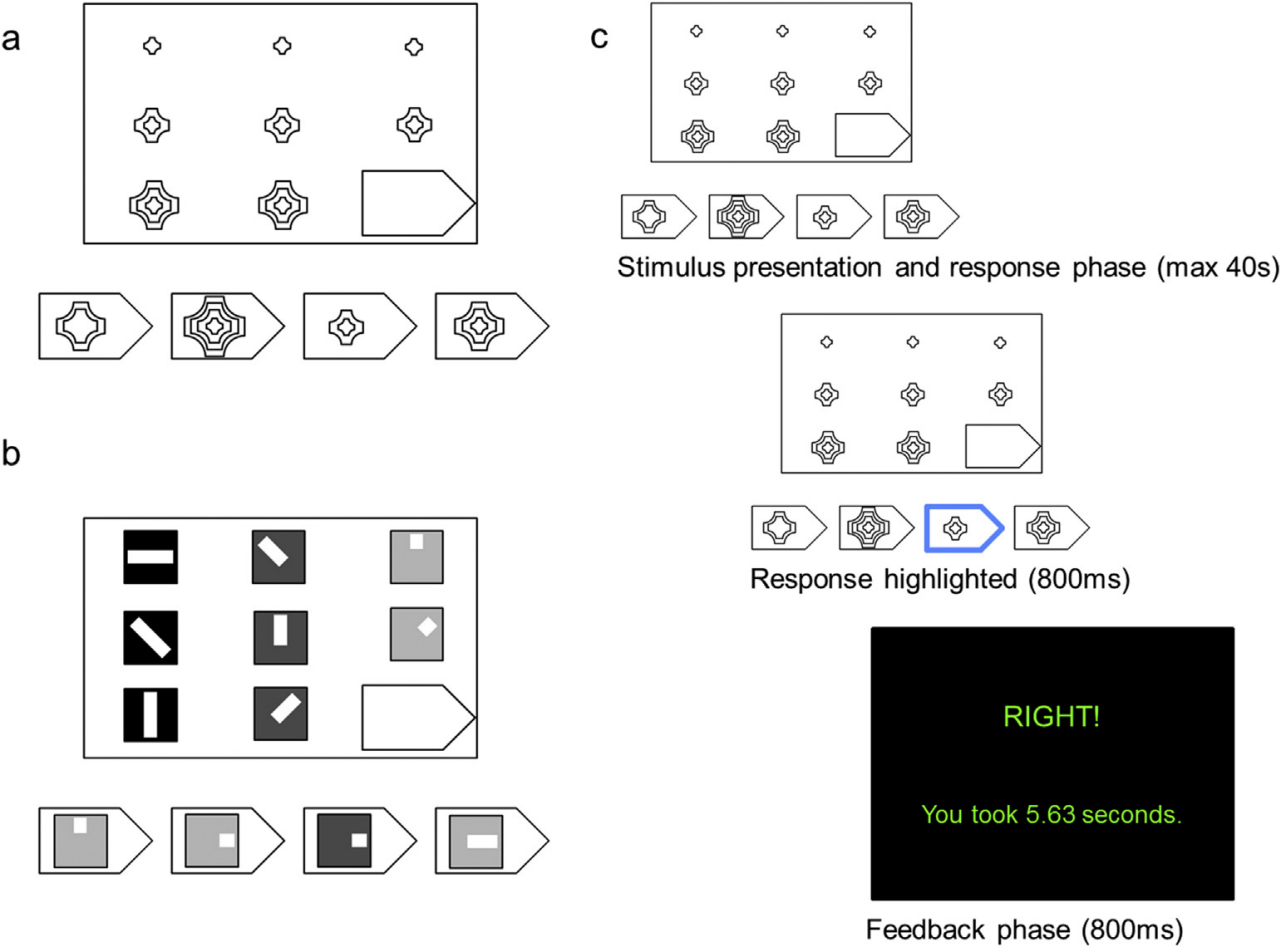
Relational Reasoning Task. a) Example of a low-relational problem: 1-relational reasoning matrix, with a vertical increase in the number of items (the correct response is the first from the right). b) Example of a high-relational problem: 3-relational reasoning matrix, with a horizontal change in colour, a horizontal change in the length of the bar and a change in rotation of the bar (the correct response is the second from the left). c) The stimuli were presented until the *participant* responded (within a maximum of 40 s). Next, *participants'* responses were highlighted in blue for 0.8 s. Finally, *participants* received feedback about their performance.

*Participants* solved problems that had the general form of the Raven's Progressive Matrices test (RPM, Raven, 1941). Similar to Crone et al. (2009), some of the reasoning problems were derived from the actual RPM test and additional equivalent problems were developed by us to obtain a sufficient number of simple and complex stimuli. As in the RPM, the problems contained a pattern or a 3 × 3 grid of stimuli in which the lower right stimulus was missing. Task-level was manipulated by changing the number of dimensions that needed to be considered to reach the correct solution. Low-relational trials included 36 1-relational or simple 2-relational matrices (see Fig. 1a). High-relational trials included 36 complex 2-relational (i.e. with permutation of the features within a row and/or a column) and 3- or more relational matrices (see Fig. 1b).

In each trial, *participants* were presented with four possible response options, and used a mouse to indicate their response (Fig. 1c). Stimulus presentation was self-paced (within a maximum period of 40 s per trial). *Participants* were instructed to respond as quickly and accurately as possible. The *participant's* choice was subsequently highlighted in blue (in the web version) for 0.8 s and followed by feedback about accuracy and RT for 0.8 s (Fig. 1c).

At the start of the testing session instructions were displayed on the screen and read out to the volunteer pairs. *Participants* then performed a practice session, consisting of two low-relational and four high-relational trials.

#### Social conditions

The three social conditions (alone, friend-present and experimenter-present) were manipulated in three different sessions, and the order of sessions was counterbalanced between *participants*. In each session the *participants* performed two tasks. Only the relational reasoning task is described here (the other – a perceptual discrimination task – will be analysed separately and described elsewhere). Sessions lasted on average 6.6 min and the whole study lasted 60–75 min.

In the friend- and experimenter-present conditions, the observer sat quietly behind the *participant* and watched and evaluated the *participant's* performance on the relational reasoning task. These two conditions thus differed only in terms of the relationship between the observer and the *participant*. *Participants* were aware that their performance would be evaluated by the observer in both social observation conditions. In the *friend-present* condition, the *observer* was instructed (in the presence of the *participant*) to follow the *participant's* performance closely by paying attention to both the accuracy and RT feedback and silently count the number of wrong responses (59/62 of the *observers* reported these correctly). Volunteers were instructed not to interact during the session. Similarly, in the *experimenter-present* condition, a female experimenter (LKW) explained that she would be watching the *participant's* performance closely by paying attention to both the accuracy and RT feedback, and silently counting the number of wrong responses. In the *alone* condition, *participants* performed the task without being observed by someone else.

Volunteers were tested in a quiet, spacious room. In all three social conditions, a student was working in a distant corner of the room facing away from the *participant*, to ascertain that volunteers were not communicating during the friend-present condition.

In each session, *participants* completed a set of 12 low-relational and a set of 12 high-relational matrices, randomly selected from a total set of 72 (3 sessions × 24 matrices = 72 matrices). These two sets were presented in an order counterbalanced between *participants*.

### Data analysis

Mean accuracy, and mean RT from correct trials, were analysed using 2 × 3 × 3 mixed-design ANOVAs with *task-level* (low-relational; high-relational) and *social condition* (alone; experimenter-present; friend-present) as within-subjects factors and *age group* (younger adolescents; older adolescents; adults) as between-subjects factor, using SPSS 19.0. Trials with RTs over 3 interquartile ranges above or below the upper or lower quartile of all trials were excluded from the analysis (15 out of 2232 trials). Significant interactions with age were followed up with separate repeated-measures ANOVAs for each age group, and post-hoc pairwise comparisons. One-way ANOVAs were employed to test for age-effects on verbal IQ, RPI and friendship quality. To investigate whether audience effects were related to individual differences in self-reported RPI, we performed correlation analyses between significant audience effects and RPI. Marginally significant results (p < 0.1) are reported in the results, but only significant results (p < 0.05) are further discussed. Reported effect sizes are partial eta-squared $\left({\eta_{\text{p}}}^{2}\right)$ for ANOVAs and ‘r’ for t-tests (Field, 2009). Analyses were repeated including *order* (one of six possible orders of the three social conditions) as a factor. These analyses showed no interaction between order and social condition. All effects observed in the main analyses remained significant.

## Results

### Accuracy

**Fig. 2:**
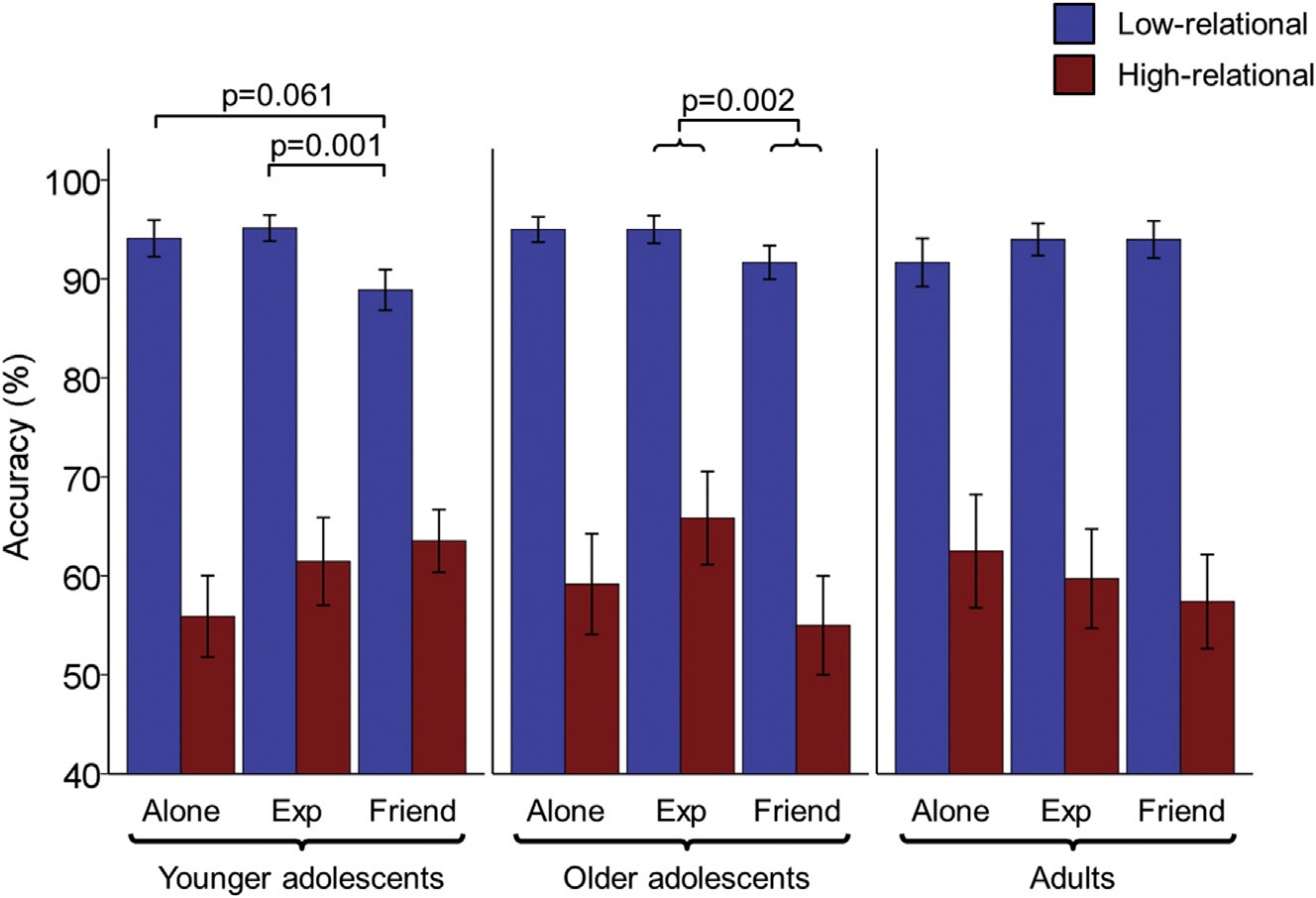
Audience Effects on Relational Reasoning Accuracy (mean ± SE). There was a three-way interaction between social condition, task-level and age group. Adults' accuracy was not affected by social condition, while older adolescents (14.9–17.8 years) showed a main effect of social condition driven by lower accuracy in the friend-present relative to the experimenter-present condition (Exp), across task-levels. Younger adolescents (10.6–14.2 years) showed a social condition × task-level interaction, driven by lower accuracy in the friend-present relative to the experimenter-present condition, and marginally lower accuracy in the friend-present relative to the alone condition, in the low-relational condition only.

There was a main effect of task-level (F(1,59) = 308.97, p < 0.001, ${\eta_{\text{p}}}^{2}\;\;$ = 0.84, Fig. 2) with higher accuracy for low-relational (93.2% ± 5.7) than high-relational trials (60.1% ± 17.5). Accuracy in low-relational trials was relatively high, thus potential audience effects might have been masked by a ceiling effect. However, accuracy was significantly different from 100% (t(61) = 9.30, p < 0.001, r = 0.77), which supports the absence of an extreme ceiling effect. There was no main effect of age group, nor an age group by task-level interaction (ps > 0.9).

The accuracy data revealed a main effect of social condition (F(2,118) = 3.49, p = 0.034, ${\eta_{\text{p}}}^{2}$ = 0.06) with *participants* responding less accurately in the friend-present condition than the experimenter-present condition overall (t(61) = 2.78, p = 0.006, r = 0.34). The other post-hoc comparisons were not significant (ps > 0.1). There was no age group by social condition interaction, nor a social condition by task-level interaction (ps > 0.25).

There was a significant three-way interaction between social condition, task-level and age group, (F(4,118) = 2.91, p = 0.024, ${\eta_{\text{p}}}^{2}$ = 0.09), which was followed up by separate social condition by task-level repeated-measures ANOVAs for each age group. The adult group showed neither a main effect of social condition nor an interaction between social condition and task-level (ps > 0.4, Fig. 2). In the younger adolescent group there was a social condition by task-level interaction (F(2,46) = 3.85, p = 0.028, ${\eta_{\text{p}}}^{2}$ = 0.14), which was further explored by separate one-way ANOVAs investigating the effect of social condition on accuracy in low-relational and high-relational trials. The effect of social condition in high-relational trials in younger adolescents was not significant (p = 0.106), however there was a significant effect of social condition on low-relational trials (F(2,46) = 4.66, p = 0.014, ${\eta_{\text{p}}}^{2}$= 0.17). This was due to a lower accuracy in the friend-present condition relative to the experimenter-present condition (t(23) = 3.89, p = 0.001, r = 0.63) and a marginally lower accuracy in the friend-present condition relative to alone condition (t(23) = 1.97, p = 0.061, r = 0.38) (Fig. 2). The alone condition and the experimenter-present condition did not significantly differ (p > 0.6). In the older adolescent group there was a main effect of social condition (F(2,38) = 4.86, p = 0.013, ${\eta_{\text{p}}}^{2}$ = 0.20) with lower accuracy in the friend-present condition relative to the experimenter-present condition across task-levels (t(19) = 3.53, p = 0.002, r = 0.63) (Fig. 2). The other post-hoc comparisons were not significant (ps > 0.1).

To summarise, no audience effect on relational reasoning accuracy was observed in adults; in contrast, older adolescents showed poorer performance when being observed by their friend relative to the experimenter, independent of task-level, while younger adolescents only showed this difference in low-relational trials.

### Response time

**Fig. 3:**
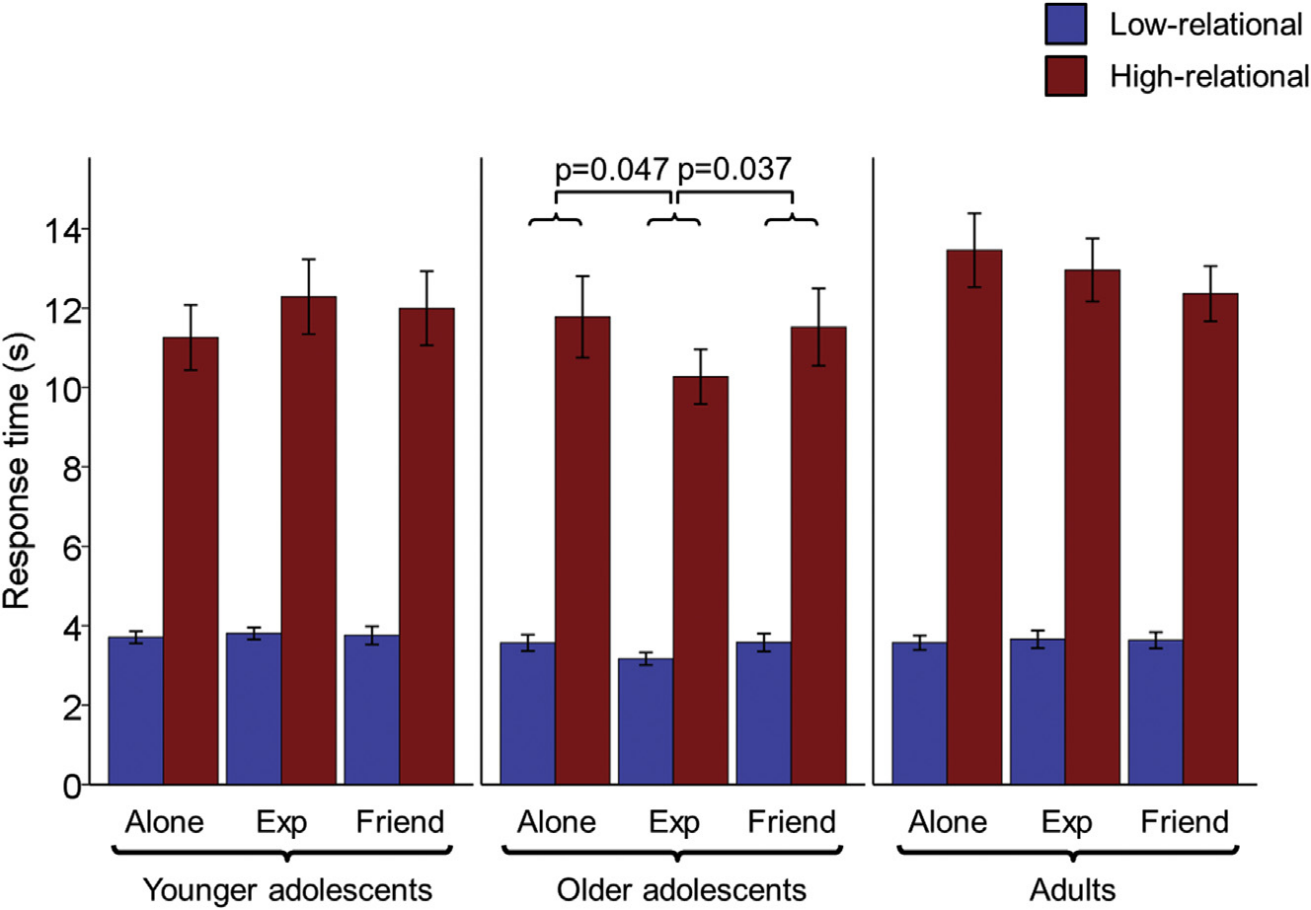
Audience Effects on Relational Reasoning RT. RT data for correct trials (mean ± SE). There was a two-way interaction between social condition and age group. Following up this two-way interaction, only older adolescents (14.9–17.8 years) showed an effect of social condition: RTs were significantly faster in the experimenter-present (Exp) relative to the friend-present and alone conditions.

There was a main effect of task-level on RT (F(1,59) = 412.88, p < 0.001, ${\eta_{\text{p}}}^{2}$ = 0.88, Fig. 3) with faster responses for low-relational (3.62s ± 0.74) than high-relational trials (11.95s ± 3.62). There was no main effect of age group, nor an interaction between age group and task-level (ps > 0.3).

Regarding social condition, there was no main effect nor a social condition by task-level interaction (ps > 0.6). The analysis revealed a two-way interaction between social condition and age group (F(4,118) = 2.84, p = 0.027, ${\eta_{\text{p}}}^{2}$ = 0.09) (Fig. 3), while the three-way interaction was not significant (p > 0.1). Separate one-way repeated-measures ANOVAs within each group were used to further explore the interaction between social condition and age. Social condition did not have a significant effect on RT in the younger adolescents or adults (ps > 0.25). However in the older adolescent group, social condition significantly affected RT (F(2,38) = 3.59, p = 0.037, ${\eta_{\text{p}}}^{2}$ = 0.16) with faster responses in the experimenter-present condition than in both the friend-present (t(19) = 2.25, p = 0.037, r = 0.46) and alone (t(19) = 2.12, p = 0.047, r = 0.44) conditions. The alone and friend-present conditions did not significantly differ (p > 0.7).

To summarise, across task-levels, older adolescents demonstrated faster RTs in the presence of the experimenter relative to the other social conditions. All reported effect sizes range from medium to large (${\eta_{\text{p}}}^{2}$: range = 0.06–0.88; r: range = 0.34–0.77) (Cohen, 1988).

### Questionnaire measures

The three age groups did not differ significantly (Table 1) in their verbal IQ scores (p > 0.25), their RPI scores (p > 0.1) or friendship quality scores (p > 0.25). To investigate whether audience effects within age groups were related to differences in RPI we correlated this measure with the significant differences in audience effects. However, none of the significant effects from the main analysis was correlated with RPI scores (ps > 0.3).

## Discussion

The current study investigated the effect of being observed by an audience, either a friend or an experimenter, on relational reasoning performance in adolescents and adults. Our study revealed three main findings. First, being observed by a friend or an experimenter affected relational reasoning in adolescents, but not in adults. Second, performance in the older adolescent group (aged 14.9–17.8) was affected by the identity of the audience: overall accuracy was lower and responses were slower when in the presence of a friend relative to the experimenter. Third, younger adolescents (aged 10.6–14.2) were less accurate when being observed by a friend relative to the experimenter in the low-relational trials only. Thus, our data suggest that audience effects on relational reasoning are critically dependent on task difficulty, the identity of the audience and the age of the participants.

### Influence of task difficulty

Audience effect studies in adults have usually found performance improvements in simple tasks and impairments in complex tasks (Bond & Titus, 1983; Zajonc, 1965). In the current study, even though task difficulty affected audience effects on accuracy performance, the analysis revealed no improvement with an audience in the low-relational condition. Few studies have manipulated task difficulty within a single experiment; thus it is hard to conclude with confidence that task difficulty determines the direction of the audience effect (Bond & Titus, 1983; Guerin, 1993). Furthermore, the fact that relational reasoning is generally considered a high-level, complex task even for problems with fewer relations to consider might also explain why we found no performance improvements with an audience.

### Developmental differences in relational reasoning

Previous studies of relational reasoning have shown performance improvements throughout late childhood and adolescence (Dumontheil et al., 2010; Rosso et al., 2004; Sternberg & Rifkin, 1979; Wendelken et al., 2011). Although not a primary aim of the study, we investigated developmental differences in relational reasoning abilities. Our data showed no evidence of age-effects on this measure. However, our task was primarily designed to maximise sensitivity to audience effects under evaluative observation: we included long response windows to permit variation in RT and provided trial-by-trial feedback that could be monitored by the *observer*. This design might have reduced the sensitivity to detect developmental differences in relational reasoning, which are usually seen when participants have a limited time to respond (Crone et al., 2009; Wendelken et al., 2011).

### Influence of the identity of the observer

A strength of the current study is that we were able to compare the effect of being observed by a peer relative to a non-peer and were thus able to control for general (e.g. distracting) effects of having someone present when performing a task. In contrast, previous studies examining peer influence effects during adolescence have usually contrasted a peer-present to an alone condition (Gardner & Steinberg, 2005; O'Brien et al., 2011; Smith et al., 2014). We thus advance this literature by demonstrating that the relationship between the *participant* and the observer, i.e. whether the observer is a peer or a non-peer, appears to be a critical factor in the audience effect in adolescence. Indeed, our data suggest that employing a non-peer condition instead of an alone condition as the control might be more sensitive to detect developmental differences in audience effects.

In addition to the identity of the observer, developmental differences in friendship quality between the *participant* and their friend might have affected the audience effects in the current study. We therefore collected a measure of friendship quality to control for potential developmental differences, but no significant differences in friendship quality between the three age groups emerged.

### Developmental differences in peer audience effects

Existing experimental studies examining the effects of peer influence in adolescence are limited in number, and have predominantly focused on the influence of peers on risky and reward-related decision-making, showing that adolescents relative to adults are especially sensitive to peer influence (Chein et al., 2011; Gardner & Steinberg, 2005; Smith et al., 2014). The age-dependent pattern of performance in the current study is consistent with findings from these studies (Gardner & Steinberg, 2005): the presence of an audience did not affect adult performance. In contrast, and also consistent with previous risk-taking studies, adolescents' relational reasoning performance was sensitive to the presence of an evaluative audience. This finding demonstrates that adolescents show a similar heightened sensitivity to peer influence on performance in a high-level cognitive task as they do when making risky or reward-related choices.

As reviewed in the introduction, based on previous experimental and questionnaire-based studies, we predicted developmental differences in the audience effect within adolescence; however it was not clear whether younger or older adolescents would show greater audience effects. The results demonstrated that relative to other age groups, older adolescents' performance was most strongly and consistently influenced by an audience. When observed by a friend relative to an experimenter they showed impairments in both accuracy and RT, across task difficulty levels. Younger adolescents' accuracy was impaired by the observation of a friend relative to an experimenter in low-relational trials only. Thus, there is stronger evidence for peer-related audience effects in the older adolescent group than the younger adolescent group. Resistance to peer influence has been shown to increase most strongly between 14 and 18 years (Steinberg & Monahan, 2007), which might have predicted greater peer audience effects in younger adolescents. However, here, the extent to which an individual *participant*'s performance was influenced by a peer audience was not correlated with *participant*'s RPI scores. Consequently, this questionnaire measure might be a better predictor of an individual's sensitivity to peer influence in the context of risky choices than of sensitivity to the presence of a peer audience when performing high-level cognitive tasks. Instead, the peer influence effects we observed might be more related to fears of being judged by a peer. Here, we did not measure this, however as reviewed in the introduction, data from another study demonstrated that 15–18 year-olds were more afraid of social evaluation than 8–11 year-olds and 12–14 year-olds (Westenberg et al., 2004). The stronger peer audience effects we observed in the older adolescents compared to the younger adolescents are consistent with heightened fear of social evaluation in older adolescents from this questionnaire-based study.

### Potential mechanisms underlying peer audience effects

While it is unclear what mechanisms underlie performance differences under evaluative observation, here we propose three potential mechanisms. First, performance changes might occur as a result of changes in arousal in the presence of others (Zajonc, 1965). Autonomic arousal is increased in adolescents relative to both children and adults when being observed by a peer (Somerville et al., 2013), which might lead to developmental differences in audience effects. Second, as reviewed above, older adolescents report elevated levels of fear of social evaluation relative to children and younger adolescents (Westenberg et al., 2004). In the presence of peers, increased fear of social evaluation might lead adolescents to spend more time mentalising about how peers judge their intellectual abilities on the basis of their task performance. This could distract *participant*s from the experimental task, resulting in an impairment of performance. Such distraction is arguably greater with a peer audience, as we controlled for general distraction caused by the presence of an observer by comparing a peer to a non-peer audience. Third, the presence of others could increase *participants'* self-awareness of potential discrepancies between their current and the ideal performance (Duval & Wicklund, 1972). This perceived discrepancy is thought to motivate performance improvement successfully in simple tasks; however in complex tasks excessive self-monitoring might impair performance. A recent study showed that, with increasing age, adolescents become increasingly aware of their own performance in a perceptual judgement task (Weil et al., 2013). In the reasoning task used in the current study, and in the presence of peers, increased self-awareness - particularly in older adolescents - may have led to a greater cognitive load due to excessive monitoring, and thus poorer performance. These three putative mechanisms should be investigated in future studies.

### Limitations and implications

There were several limitations of our study. To maximise the homogeneity of the sample, our study included female *participant*s only. Future studies should address whether similar audience effects in adolescence are found in male participants, and also whether audience effects differ for different-sex peers. Both the adolescent and adult volunteers were from high-achieving academic backgrounds. Although this benefitted the homogeneity of our sample, it limits the generalizability of our study. Future studies should test whether similar audience effects can be found in more academically diverse samples. Our research extends previous studies on peer influence by including a second social observation condition, allowing us to control for general effects of being observed. However, while the familiarity of the peer and non-peer was matched in adolescents and adults, the age difference between the peer and non-peer was not. Consequently, our experimental design does not allow us to conclude whether the difference in age or the difference in familiarity between the peer or non-peer observer underlies the observed pattern of audience effects. This could also be addressed in future studies that fully balance these two factors of age and familiarity. Finally, the study draws on a relatively small sample size and further replications are needed.

Lessons, exams and homework are often carried out in the presence of other people – students, teachers, siblings and/or parents. In these situations, adolescents' performance is often either implicitly or explicitly evaluated by others. Although we did not test for audience effects in an educational setting, we demonstrated audience effects on relational reasoning, a cognitive capacity which is a critical for children's learning and related to academic knowledge, reading and mathematics performance (Ferrer et al., 2009; Träff, 2013). Our data suggest that performance on a relational reasoning task in adolescence is sensitive to the identity of the person observing and evaluating their performance. Further work will be needed to identify how a collaborative or competitive context, as typically occurs in schools, would affect the peer audience effect during adolescence.

## Figures and Tables

**Table 1:** Age, Verbal IQ, Resistance to Peer Influence and Friendship Quality scores. Verbal IQ of the *participant* groups were estimated with the vocabulary subtest of the WASI (Wechsler, 1999). *Participants* completed the Resistance to Peer Influence questionnaire (RPI, Steinberg & Monahan, 2007). Volunteers completed the McGill Friendship Questionnaire–Friend's Function (MFQ-FF, Mendelson & Aboud, 1999) and for each volunteer-pair a combined score of *participant* and *observer* reported Friendship Quality was generated.

	Age group	Age				Verbal IQ^a^			RPI^c^			Friendship Quality^d^		
		n	Range	Mean	SD	n	Mean	SD	n	Mean	SD	n	Mean	SD
*Participant*	Younger Adolescents	24	10.6–14.2	12.8	1.0	24	121.3	8.4	24	2.9	0.4	23^e^	6.7	0.7
	Older Adolescents	20	14.9–17.8	16.4	1.0	20	116.7	10.0	20	2.9	0.5	20	6.9	0.5
	Adults	18	21.8–34.9	27.3	3.7	16^b^	117.6	10.7	18	3.1	0.3	18	6.5	0.9
*Observer*	Younger Adolescents	24	10.9–14.6	13.1	1.1									
	Older Adolescents	20	14.8–17.6	16.3	0.9									
	Adults	18	22.4–31.4	26.5	2.7									

a p > 0.25.

b Verbal IQ was not collected for two adult *participants*.

c p > 0.1.

d p > 0.25.

e Friendship Quality was not collected for one younger adolescent *observer*.
